# Quantification of Residual Perfume by Py-GC-MS in Fragrance Encapsulate Polymeric Materials Intended for Biodegradation Tests

**DOI:** 10.3390/molecules25030718

**Published:** 2020-02-07

**Authors:** Giulio Gasparini, Sarah Semaoui, Jessica Augugliaro, Alain Boschung, Damien Berthier, Markus Seyfried, Frédéric Begnaud

**Affiliations:** 1 Firmenich SA, Corporate R&D Division, Route des Jeunes 1, 1211 Genève, Switzerland; 2 Union Française du Commerce Chimique, 75000 Paris, France

**Keywords:** microcapsules, perfume quantification, Py-GC-MS

## Abstract

Perfume encapsulates are widely used in commercial products to control the kinetic release of odorant molecules, increase storage stability and/or improve deposition on different substrates. In most of the cases, they consist of core-shell polymeric microcapsules that contain fragrance molecules. A current challenge is to design and produce polymeric materials for encapsulation that are both resistant and non-persistent. The selection of such eco-friendly formulations is linked to a deep understanding of the polymeric material used for encapsulation and its biodegradation profile. To collect this information, pure samples of capsule shells are needed. In this article we present an innovative quantification method for residual volatiles based on pyrolysis-GC-MS to enable validation of sample quality prior to further testing. The presented analytical method also led to the development of a robust and comprehensive purification protocol for polymers from commercial samples. Standard techniques are not suited for this kind of measurement due to the non-covalent embedding of volatiles in the 3D structure of the polymers. We demonstrated the confounding impact of residual volatiles on the estimated biodegradability of fragrance encapsulates.

## 1. Introduction

Perfumes are ubiquitous additives in consumer goods like laundry, home and personal care products. They are essential not only for technical reasons (e.g., to cover unwanted odours), but also to elicit positive emotions and support brand recognition [1]. Flavour and fragrance companies continuously work on the discovery of new ingredients to enlarge the palettes available to perfumers, allowing them to generate and refine their own signature creations. Technologies are also intensively used to enhance consumer experiences by fine tuning perfume release through state-of-the-art delivery systems. In this quest for continuous improvement, sustainability is a main focus for almost all companies. This approach results in a commitment to market eco-friendly ingredients and technologies [2,3].

Different delivery systems have been developed across the years, notably in order to control the kinetic release of odorant molecules, increase storage stability and/or improve deposition on different substrates [4,5,6,7]. Core-shell microcapsules are the most used, thanks to the possibility of perfume release as a result of an external stimulus, e.g., mechanical rubbing [8,9,10], light exposure [11,12,13] temperature [14,15] or pH change [16,17,18]. They consist of a spherical shell of cross-linked polymer (e.g., polyurea, polyurethane, melamine-formaldehyde, polyamides, etc.) that contains fragrance molecules and protects them from evaporation, dispersion and reaction with other ingredients of the final product. A current challenge is to design and produce polymeric encapsulation materials that are both resistant to environmental damage and non-persistent. They need to survive the harsh conditions in consumer products (extreme pH, presence of active species, high concentration of surfactants, etc.) and potential damage in-use but also to undergo rapid degradation to avoid accumulation in the environment once they have fulfilled their function.

The need for eco-friendly fragrance encapsulates is in accordance with the European Chemicals Agency (ECHA) which is moving toward increased control of polymers under Registration, Evaluation, Authorisation and Restriction of Chemicals (REACH). In the proposed Annex XV restriction report [19], ECHA considers restricting the use of intentionally added microplastic particles (primary microplastics) in consumer or professional use products of any kind. Based on their dimensions (between 1 nm and 5 mm as per the ECHA definition, still subjected to potential revisions), microcapsules used in the perfumery industry for home and personal care applications by all the fragrance houses may be concerned. However, the release of fragrance encapsulate polymeric debris is minimal compared with microplastics present in the environment as a result of fragmentation of bigger objects or synthetic fibres (secondary microplastics). Moreover, fragrance encapsulates have never been identified in aquatic litter (more than 98% of similar materials were shown to be removed in the sludge of wastewater treatment plants [20]). Nevertheless, enormous efforts are currently being made to develop microcapsule walls that are eco-friendlier [2]. In this regard, biodegradation tests are meaningful to assess the degradability of prototypes and therefore guide the selection of future eco-friendly formulations.

Organisation for Economic Co-operation and Development (OECD) guidelines provide benchmarks on how to perform biodegradation tests for pure ingredients [21], but discussions are still ongoing regarding polymeric materials. Some ISO norms do exist for plastic materials, but so far these materials were mostly evaluated based on their ability to degrade in composting units [22]. Despite there being no official position on polymers yet, it seems meaningful that the material to be tested should be comparable in terms of chemical and physical composition to the one that will reach the environment, as these parameters may impact the degradation behaviour.

Fragrance encapsulates are usually commercialized by the major fragrance houses as suspensions in water, called slurries, containing not only the core shell microcapsules with the perfume, but also different deposition aids, preservatives, emulsifiers, etc., in non-covalent interaction with the shell. For this reason, it is not possible to perform direct measurements (e.g., biodegradation studies) on the polymeric shells of microcapsules without proper preparation of commercial samples. The presence of unbonded biodegradable residuals not related to the polymer would impair the reliability and the trustiness of the results, leading to measured levels of biodegradability which are inaccurate and misleadingly high. It is therefore necessary to apply a purification protocol that yields the covalently bonded microcapsule polymeric shell material in high purity and without any modifications to avoid misleading results. The subsequent quantification of residuals in shell samples is therefore crucial to confirm if the prepared shells are representative of the polymeric material. So far, standardized methods for sample preparation do not exist and, when biodegradation results are given, little or no data on the characterization of residuals is reported. This lack of references generates a lot of confusion in the flavour and fragrance field and may potentially lead to major misinterpretations of the evaluation of the eco-friendliness of new perfume encapsulates.

In the present study, we introduce and discuss an innovative analytical method based on pyrolysis-GC-MS (Py-GC-MS) to quantify the residual volatile compounds observed to be frequently responsible for unreliable biodegradability results in microcapsule shell samples. This method allowed the development of a robust and comprehensive purification protocol that ensured the quality of polymeric material for biodegradation testing from commercial fragrance encapsulates sources. We aimed to obtain materials with less than 4%_*w*/*w*_ of residual perfume. This threshold was set considering that the accepted variability of biological experiments is higher than the potential contribution of the residual perfume as a carbon source to the final result. Indeed, this is especially true considering that the typical biodegradation tests conducted on pure ingredients (OECD guidelines) tolerate a relative difference up to 20% between two measurements performed simultaneously on the same product [21]. The Py-GC-MS technique was necessary to access and measure the perfumery raw materials that may be both trapped physically in the mesh of the polymeric network and adsorbed reversibly on the solid. To our best knowledge, this is the first time that quantification of volatiles in powder has been conducted by Py-GC-MS.

## 2. Materials and Methods

### 2.1. Fragrance Oil Composition

The fragrance oil used to generate the core shell microcapsules consisted of a mixture of five fragrance ingredients, present in equal amounts (*w*/*w*): (+/−)methyl-2,2-dimethyl-6-methylidene cyclohexanecarboxylate (Romascone^®^, CAS: 81752-87-6, (Linear Retention Indices) LRI = 1172), (1RS,2RS) 2-(2-methyl-2-propanyl) cyclohexyl acetate (Verdox^TM^, CAS: 88-41-5, LRI_main_ = 1284; LRI_minor_ = 1303), cis or trans 4-tert-butyl-1-cyclohexyl acetate (Lorysia^®^, CAS: 1900-69-2, LRI_trans_ = 1320; LRI_cis_ = 1356), (+/−)-3-(4-isopropylphenyl)-2-methylpropanal (Cyclosal, CAS: 103-95-7, LRI = 1434), (Z)-2-phenyl-2-hexenenitrile (Salicynile^®^, CAS: 6519-09-1, LRI = 1449). All LRIs were calculated on a non-polar GC column.

### 2.2. Microcapsule Preparation

Core shell microcapsules containing fragrance oil were prepared according to literature procedures [10,12,23].

Sample 1: Ambergum^TM^ 1221 (Aqualon) (15.0 g) was added to water (30 mL) containing 4.5 g of resin. The resin was composed of 9.3 g (74 mmol) of 1,3,5-triazine-2,4,6-triamine (melamine), 13.3 g (222 mmol) of urea, 12.8 g (74 mmol) of 2,2-dimethoxyacetaldehyde (2,2-dimethoxy ethanal or DME) and 42.9 g (296 mmol) of oxalaldehyde (glyoxal). The reaction mixture was stirred for 30 min at RT, then a solution of fragrance compounds (20 g), containing 1.67 g of polyisocyanate (Takenate^®^ D-110N, Mitsui Chemicals), was added. The resulting mixture was emulsified at RT for 2 min using an Ultra-Turrax^®^ disperser (24,000 rpm). An aqueous solution of Alcapsol^®^ 144 (0.4 g) was added. The emulsion was stirred with an anchor-shaped paddle at 40 °C for 1h, then the temperature was increased to 60 °C and maintained for additional 1 h, yielding a white dispersion of core shell microcapsules (slurry).

Sample 2: 11.43 g of Melamine-formaldehyde resin (Urecoll SMV, BASF), colloidal stabilizer (59.2 g, 20%*_w_*_/*w*_ in water, Poly(acrylamide 20%, acrylic acid 80%) sodium salt and water (376 g) were introduced into a 1000 mL reactor at room temperature. The resulting solution was mixed with an anchor-shaped paddle and 1.8 g of acetic acid were added, to bring the pH value to 5.1. The reaction mixture was stirred at 45 °C for 1 h. Fragrance oil (270 g) and polyisocyanate (5.3 g, Takenate^®^ D-110N, Mitsui Chemicals) were added and the resulting mixture was emulsified with an Ultra-Turrax^®^ at 13,500 rpm for 2 min. The emulsion was stirred at 80 °C for 2 h. A total of 56 g of a solution 50%*_w_*_/*w*_ of ethylene urea were added and the emulsion stirred for additional 1 h at 80 °C. The dispersion was cooled to RT and the final pH was corrected to 6.5 using NaOH 30%_*w*/*w*_ in water. Core shell microcapsules were obtained as a white dispersion.

Sample 3: A solution of poly (vinyl alcohol) PVOH 18-88 in water (510 g, 1%_*w*/*w*_) was added to a 1000 mL reactor. A solution of polyisocyanate (50 g, Takenate^®^ D-110N, Mitsui Chemicals) in perfume oil (380 g) was added to the reactor and the reaction mixture was stirred with an Ultra-Turrax^®^ at 17,500 rpm for 4 min. The resulting emulsion was mechanically stirred at 350 rpm with an anchor-shaped paddle. A solution of guanidine carbonate (8.8 g) in water (50 g) was added dropwise over the course of 1 h and the reaction mixture was then warmed up to 70 °C over the course of 1 h. The reaction mixture was stirred at 70 °C for two additional hours and finally cooled down to RT to afford a white dispersion.

Sample 4: In a round bottom flask, 2-oxoacetic acid (5.1 g, 50%*_w_*_/*w*_ in water, Sigma-Aldrich) was added to a solution of carboxymethyl cellulose (Ambergum^TM^ 1221, Aqualon) in water (400 g, 4%*_w_*_/*w*_). A solution of polyisocyanate (19 g, Takenate^®^ D-110N, Mitsui Chemicals) in perfume oil (250 g) was added to the flask and the reaction mixture was stirred with an Ultra-Turrax^®^ at 17,500 rpm for 2 min. The resulting emulsion was mechanically stirred at 300 rpm with an anchor-shaped paddle at 45 °C for 1 h, then at 60 °C and finally at 80 °C for 2 h. A solution of copolymer of acrylamide and (3-acrylamidopropyl)-trimethylammonium chloride (Salcare^®^ SC60, Ciba) at 3%*_w_*_/*w*_ in water (340 g) was added to the dispersion and the reaction mixture was stirred at 80 °C for 1 h. The mixture was finally cooled down to RT, affording a white dispersion.

### 2.3. Purification Protocol for Shell Preparation

**Figure 1:**
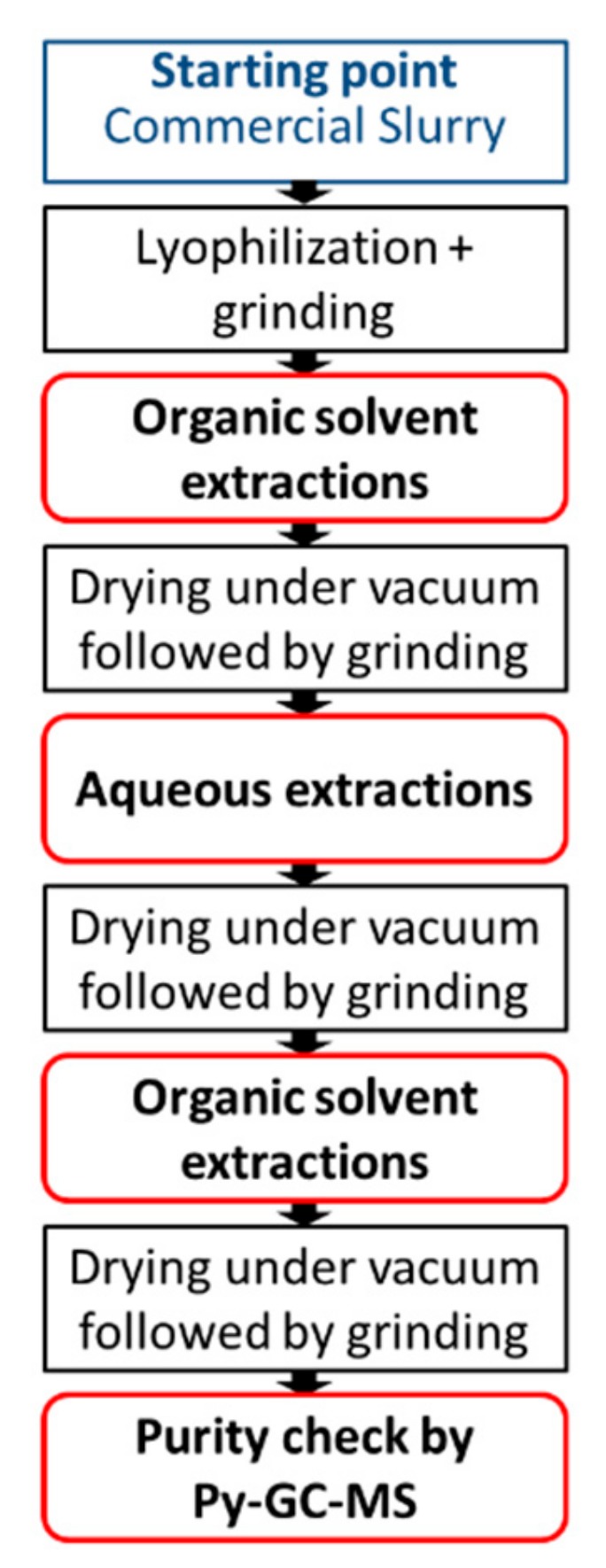
Extraction protocol for microcapsule shell preparation.

The following protocol was developed to prepare pure capsule shell material for further analysis (e.g., biodegradation studies, environmental effects, toxicology, etc.). The objective was to remove all the non-covalently bonded and as well any residual volatile material. The general procedure is represented in Figure 1.

Detailed example of the procedure: commercial slurry (700 mL) containing Sample 3 fragrance encapsulates was lyophilized. The resulting solid (264.0 g) was homogenized using a ball mill (Retsch mixer mill 400, 5 min; 30 Hz; 35 mL vial, 3 balls × 5 mm ball diameter), to yield a residue with an average particle size between 1.5 and 5 µm (measured by flow particle image analysis). The resulting paste (fragrance oil + polymeric shells) was suspended in 1.6 L of ethyl acetate and the mixture was stirred for 1 h at RT. The solid was collected by filtration under vacuum over a gooch filter crucible (porosity 4). The solid was then recovered and dried under vacuum (10 mBar) at 50 °C until the weight of the polymer, monitored by gravimetry, was constant. Thermogravimetic analysis was performed on a TGA/SDTA851e (Mettler Toledo AG, Switzerland) instrument, equipped with a microbalance (1 µg accuracy) and a 35 mL oven. The sample (about 12 mg in a 70 µL aluminium crucible) mass loss was measured under constant nitrogen flow (20 mL/min) at temperatures from 25 to 50 °C (rate 5 °C/min) and then at 50 °C for four hours. The extraction step was repeated a total of five times, yielding at the end of the organic extractions, 36.8 g of a white powder.

The powder (35.0 g) was suspended in mQ water (0.5%*_w_*_/*w*_) and stirred for 24 h. The solid was recovered by centrifugation (filtration was not feasible due to the colloidal behaviour of the suspension, resulting in clogging of the filters). The solid was washed twice. After centrifugation, the resulting paste was collected and dried for 2.5 days at RT, then under vacuum (10 mBar) at 50 °C overnight, yielding 21.4 g of white solid, homogenized by blade milling (IKA Tube Mill, 5 min; 20,100 rpm). The recovery yield (61%) of this washing step is ascribed to the difficulty of separating the water phase from the polymer rather than the amount of residual water soluble non-bonded materials.

The solid obtained from water extraction (20.9 g) was extracted an additional five times with ethyl acetate as described before. It was dried under vacuum (10 mBar) at 50 °C overnight to obtain 19.2 g of white powder.

### 2.4. SPME-GC-MS Measurements

Analyses were carried out using a 7890 Gas Chromatograph (Agilent, Santa Clara, California, USA) coupled to a MS detector (5975C inert XL MSD, Agilent). The analytical system was driven using ChemStation software (Agilent, version E02.02.1431). The polymer sample (50 mg) was accurately weighed in a 10 mL vial. Sample extraction and injection were automated using a CTC CombiPal autosampler (Zwingen, Switzerland) equipped with a SPME fiber holder and a temperature controlled six-vial agitator tray and driven by Cycle Composer software (ver. 1.4.0). A dedicated method was developed in-house to automate both the sample and external standard (methyl n-octanoate 1% in mineral pump oil, Edwards 4) extractions and injection, limiting the experimental variability of the sampling and extraction processes. PDMS fibers (Supelco, 7 µm) were conditioned before use according to the supplier recommendations. The sample was incubated for 60 min at 60 °C and exposed to a SPME fiber for 10 min; subsequently, the SPME fiber was exposed for 20 s to the external standard headspace, kept at 50 °C. Once loaded with the sample and the external standard, fibers were thermodesorbed in the GC injector inlet at 250 °C in splitless mode and a split 1/200 opened after 30 s. Purified helium was used as the carrier gas. Separation was performed on DB-1ms fused silica column (30 m × 0.25 mm × 0.25 µm, Agilent). The injector was set at 250 °C, and constant flow mode was selected for all analyses. The column was initially set at 80 °C for 1 min and then ramped at 15 °C/min to 222 °C then ramped at 15 °C/min to 260 °C, holding for 1 min; giving a total run time of 12.9 min. Helium flow was 1 mL/min. The MS was operated in SIM mode (ions: 74 amu for external standard methyl n-octanoate, 82 amu for Verdox^TM^ and Lorysia^®^, 122 amu for Romascone^®^, 129 amu for Salycinile^®^, 138 amu for Cyclosal), dwell time 80 ms.

### 2.5. Py-GC-MS Measurements

Analyses were carried out using a Thermal Desorption Unit TDU coupled with an Automated Pyrolysis Module and a Cooled Injection System CIS (Gerstel GmbH, Germany) installed on a 6890 Gas Chromatograph (Agilent, USA) coupled to a MS detector (5975C, Agilent). Sample injections were performed using an automated system comprising a CTC CombiPal autosampler (Zwingen, Switzerland) equipped with a pyrolysis tube holder. Between 50 and 150 µg of solid samples were weighed in closed bottom venting slit quartz microvials (Gerstel GmbH, Germany) using a precision weight scale (Mettler Toledo AT20). The vials were placed in the pyrolyser, that is inside the thermal desorption unit (TDU). The latter follows the pyrolysis temperature from 90 to 350 °C to stabilize the heating ramp. Between 100 and 350 °C volatile compounds in the sample were thermo-desorbed, then the sample was thermally degraded. The temperature was increased at 5 °C/s to 700 °C under helium (0.8 mL/min). The volatiles were conveyed through a short connection to the CIS, maintained at 300 °C and 280 °C, respectively. The volatiles were then focused on the top of the GC column (DB-5ms, 30 m × 0.25 mm ID, film thickness 0.25 µm, Agilent), since the starting temperature in the oven was set at 50 °C. The GC injector operated in split mode (100:1), and the temperature was maintained at 50 °C for 0.5 min, then ramped at 15 °C/min to 320 °C, hold 2 min, giving a total GC run time of 20.5 min. The compounds were detected using a single quadrupole mass detector (5975C, Agilent) working both in scan range (from 10-500 amu) and in SIM mode (ions: 82 amu for Verdox^TM^ and Lorysia^®^, 122 amu for Romascone^®^, 129 amu for Salycinile^®^, 138 amu for Cyclosal, 183 and 264 amu for internal standard 1,2-dibromo-4,5-dimethylbenzene).

### 2.6. Preparation of Standard Solutions

Calibration solutions containing the same quantity of internal standard (IS, 1,2-dibromo-4,5-dimethylbenzene, CAS: 24932-48-7, LRI = 1173), and perfume oil at different concentrations were made. The weight percentage of perfume oil was defined compared to the quantity of the shell present in the micro vial for analysis. (due to the impossibility to reproducibly weigh such low masses). Six calibration solutions of 10%, 3%, 1%, 0.3%, 0.1% and 0.03% of perfume oil were made in ethyl acetate containing 0.1 mg/mL of IS. 5 µL of each calibration solution were placed in pyrolysis vials and analysed immediately to avoid evaporation.

The ratios between the peak area of each perfume ingredient and the internal standard were plotted against the perfume amount. Linear regression was used to obtain the calibration curves for each component and, by summing all contributions, also for the total amount of perfume. The resulting equations and corresponding r^2^ values are reported in Table 1.

### 2.7. Standard Additions to Py-GC-MS Samples

The shell samples (approximately 50 µg) were accurately weighed directly in the pyrolysis microvials. Due to the low masses to be manipulated, a weight distribution ranging between 40 µg to 120 µg was obtained. The volume of perfume standard solution added was adapted in order to have samples ranging between 0.5%*_w_*_/*w*_ to 10%*_w_*_/*w*_ compared to the solid, without exceeding the maximum volume admitted by the venting slit (10 µL). As a result, the amount of IS was not constant since adapting the volume changed the overall quantity of IS. This was taken into account by multiplying the internal standard peak area by the ratio between the actual and nominal volumes of solution used (5 µL). Standard addition quantifications were performed on three aliquots of the same sample.

### 2.8. Biodegradation Experiments

Purified shell materials were tested for biodegradability using an adaptation of the OECD 301F guideline [21], as described previously [24].

Purified shell material was dispersed in mineral media (pH 7.4) at 0.5%*_w_*_/*w*_ and stirred overnight, before being mixed into 100 mL of inoculated culture medium (mineral solution) for a concentration corresponding to 100 mg/L of dry test material and 30 mg/L of dry sludge inoculum. The test system consisted of a sample flask sealed with a sensor head/CO_2_ trap. The samples were stirred for the duration of the study with a magnetic stirrer. The test was conducted in diffuse light at a temperature of 22 °C +/− 1 °C, and the oxygen consumption was automatically calculated. The total amount of oxygen required to oxidize the chemical completely was calculated from elemental analysis results and expressed as mg oxygen required per mg test item.

## 3. Results and Discussion

Prior to the setup of a rigorous purification procedure and the related quantification method for residual volatiles, some preliminary biodegradation tests on encapsulating shell materials were conducted. We used commercial slurries that were extracted to recover the solid polymeric material. The solid obtained after lyophilisation of the slurry was washed with ethyl acetate and water until an assessment of the residual perfume oil content by TGA revealed a weight loss lower than 1% at 50 °C. Following this procedure, the samples independently prepared from the same formulation yielded unacceptable biodegradation variability, ranging from 5 to 36%. This was particularly striking when the same extracted material, kept at room temperature, was tested after up to six months and resulted in significantly different results. Despite the reference material giving the expected biodegradation (indicating that the validity criteria of the biodegradation test were fulfilled at all times), the results for the aged shells were lower by more than 25% compared to the results obtained during earlier tests. This value was high even when considering the variability of biodegradation tests with inoculum from wastewater treatment plants. Therefore, we postulated the presence of considerable amounts of residual volatiles, the main biodegradable constituents of the capsules, to explain this variability. It was also a clear indication that TGA measurements were inappropriate to determine the real amounts of volatiles remaining in the shells.

### 3.1. Purification Protocol for Residual Volatiles Removal

**Figure 2:**
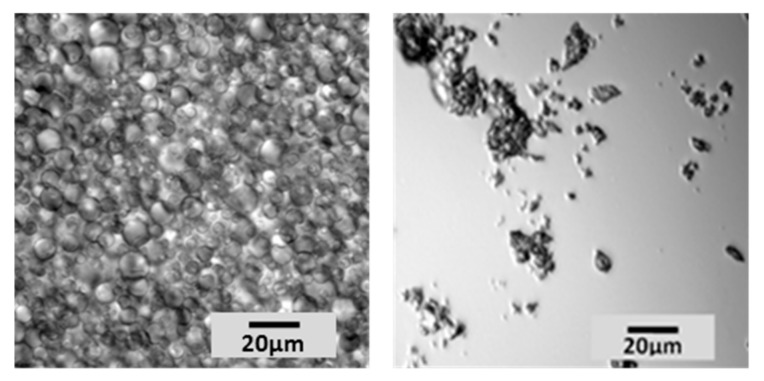
Microcapsules obtained after slurry lyophilisation (**left**) and the corresponding polymeric shells obtained after ball milling (**right**).

Core shell microcapsule polymers were obtained from commercial slurry. Water, representing about 60% of the total mass of the starting material, was removed by lyophilization. The freeze-drying process did not impact the characteristics of microcapsules, which remained intact and filled with perfume. The solid was recovered and submitted to grinding, to break the microcapsules (that become brittle once dried) and release the encapsulated volatiles. Two different types of grinding were applied (ball milling or blade blending), obtaining similar results and no traces of intact microcapsules were detected by light microscopy using either approach (Figure 2).

The removal of perfumery raw materials and all non-covalent bonded substances from the polymer constituting the shells was achieved by sequential extractions of the solid material with ethyl acetate and water, grinding the solid between each step. The first series of extractions with organic solvent removed the majority of perfume. Indeed, the quantity of recovered solid was monitored after each extraction (total of 5) by gravimetry, until constant (with a margin of error of 1%), yielding between 10% and 15% of the total weight of the solid obtained after lyophilization. An extraction step with water was necessary following the organic extractions steps to eliminate all water-soluble entities that may be trapped within the polymer shells and may hamper perfume release. Considering the initial amounts of water-soluble components in the formulations, especially if compared with the quantity of encapsulated perfume, major impacts on further analyses of shells were not expected. The solid was extracted twice with water, with the formation of colloidal dispersions. It was not possible to follow the extraction procedure by gravimetry due to the practical difficulties of recovering the solid in a quantitative way. A final series of extractions with ethyl acetate aimed at minimizing the residual volatiles that may still have been present in the polymeric substrate.

### 3.2. Tentative Quantification of Residual Perfume by SPME-GC-MS

The quantification of the residual perfume present in the solid material at the end of the extraction process was attempted using solid phase micro extraction (SPME) coupled with GC-MS analysis. This technique has the advantages of being straightforward and rapid and of requiring standard instrumentation. The principle was to measure and quantify, by GC-MS, the ingredients that were released from the solid when submitted to gentle heating in controlled conditions. It was possible to perform quantification measurements by applying a methodology based on the use of an external standard (methyl n-octanoate) dissolved in a non-volatile liquid (silicone oil), as described by Pawliszyn et al. [25].

The procedure to perform the analysis consisted of exposing the SPME fibre for a defined period of time to a known amount of solid sample contained in a 10 mL vial and maintained at a fixed temperature. The fibre was then exposed to the headspace of a vial containing the external standard and finally thermodesorbed in a GC-MS injector. Several parameters of the method were studied and optimized in order to find the best compromise between accuracy, time needed for the analysis and ease of use including fibre type, incubation time, extraction time and calibration curve for standard addition.

It was first verified that analysing spiked polyurea/polyurethane polymer powder with perfume using SPME led to acceptable calibration curves (r^2^ = 0.9898 measured on four calibration points ranging from 0.01%*_w_*_/*w*_ to 0.1%*_w_*_/*w*_). The tested polymer had a composition and characteristics very similar to those of the microencapsulation shells of concern without did not contain perfume oil.

**Figure 3:**
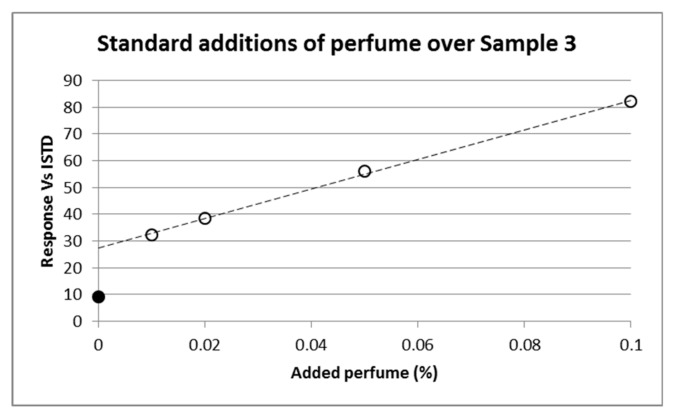
Standard addition to Sample 3 shells, measured by SPME-GC-MS. Filled circle represents the point of zero addition, clearly deviating from the linear perfume adsorption behaviour (dotted line) obtained by standard addition.

The model perfume was constituted of five perfumery raw materials, two of which being composed by two isomers, for a total of seven volatile molecules. Quantification of residual perfume in the shells using the calibration curve generated by standard addition and SPME gave surprisingly low values. In order to validate the method, standard additions were performed directly on the microcapsules shell material. This evidenced the non-linearity of the curve, in which the point corresponding to zero-addition was clearly an outlier (Figure 3). We hypothesized that the perfumery molecules in the polymeric shell were embedded (but not covalently linked) into the polymeric frame during the formation of the microcapsule by emulsion polymerization and were released at a significantly slower pace than the ones present on the surface; therefore, a comprehensive treatment to release them was necessary. This was not the case with the standard addition, because the volatile molecules were adsorbed but did not intimately penetrate the bulk polymer framework.

### 3.3. Quantification of Residual Perfume by Py-GC-MS

**Figure 4:**
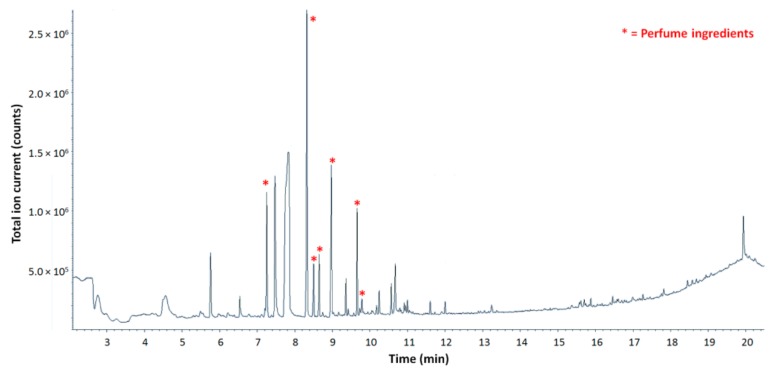
Typical pyrogram obtained for samples submitted to Py-GC-MS analysis (Sample 3). The chromatogram peaks corresponding to perfume ingredients are highlighted in red. The other peaks present correspond to pyrolysis products of the polymeric shell.

In Py-GC-MS the sample is thermally degraded to produce volatiles that are separated in the GC column and detected by a MS detector. The thermal degradation of the sample takes place in an inert atmosphere at temperatures ranging up to 1000 °C. The heating induces the dissociation of chemical bonds within the molecules [26]. In a polymeric sample, such as microcapsule shells, the thermal degradation results in the deconstruction of the crosslinked molecular network. If the temperature increase is gradual, volatiles that may be embedded in the polymeric frame can be released before the actual pyrolysis (thermal degradation of the whole sample with the production of free radicals) takes place. It is therefore possible to detect perfumery molecules together with the pyrolysis products of the polymeric shell, providing that network destructuration occurs before thermal degradation of the targeted molecules. In the pyrograms obtained from all samples submitted to analysis that contained a model perfume made of five raw materials, no traces of degradation or further reactions of perfumery molecules were detected, confirming their complete and unaltered release from the matrix (Figure 4). 

**Figure 5:**
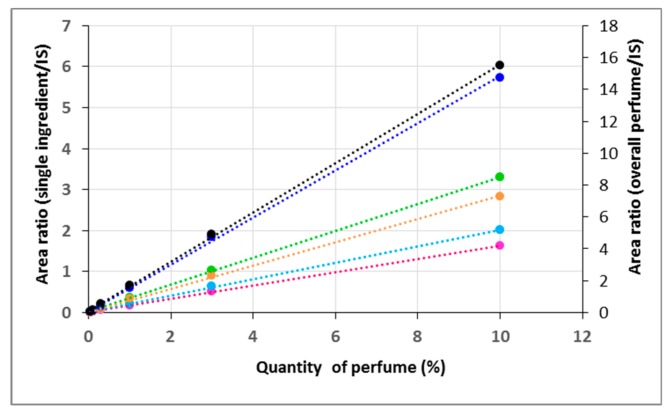
Example of calibration curves obtained by Py-GC-MS for Romascone^®^ (pink), Verdox^TM^ (green), Lorysia^®^ (light blue), Cyclosal (orange), Salicynile^®^ (blue) and the corresponding overall perfume (black) for Sample 1.

A calibration curve using solutions of pure model perfume was established by Py-GC-MS since the very small amount of solid material required for analysis (40 to 150 µg) prevented the application of the same polyurea/polyurethane polymer approach as described before. Such small amounts generate very poor weight reproducibility. The use of an internal standard (4,5-dibromo-o-xylene) was mandatory to increase accuracy by compensating for the overall variability. The calibration curves obtained for single volatiles or for the whole perfume (obtained by the sum of the contribution of each single perfume ingredient) showed excellent linearity (Figure 5).

**Figure 6:**
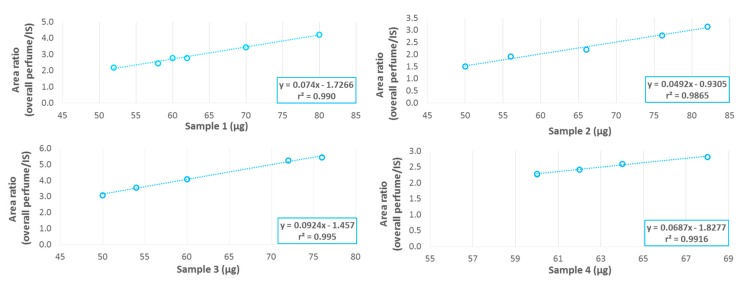
Py-GC-MS repeatability studies for Sample 1 (upper left), Sample 2 (upper right), Sample 3 (lower left) and Sample 4 (lower right). At least five independent replicates were recorded.

The perfume to shell mass ratio repeatability was studied on different shell formulations to evaluate the robustness of the measurements. Since sample weighting was highly variable due to the µg-range of sample needed for Py-GC-MS, it was not possible to perform the analyses on exactly the same sample masses. Consequently, the repeatability measured included the impact of different weights. Repeatability can be observed in the plots of the total area of ingredients (i.e., total perfume) normalized by the area of the internal standard, versus the mass of shells analysed. At least five independent replicates were measured for each formulation (Figure 6), obtaining excellent linearity (r^2^ ≥ 0.99) for all the four materials tested. Repeatability, estimated using the relative standard deviation of perfume quantification in the shells, was in the range of 7 to 9%. Thanks to this quantification technique, the liquid–solid extraction steps with organic and aqueous solvents were optimised, ensuring that the residual perfume amount was below the expected 4%*_w_*_/*w*_ limit for all the polymers tested, representing the majority of the types of chemistry present in commercial products. The extensive extraction of the solid materials with organic and aqueous solvents was necessary since, based on the external calibration curve measured; some traces of residual perfume were still present but in every case within the 4%_*w*/*w*_ limit, see below. The quantification of residual volatiles is a crucial need for the development of a robust sample purification method and for checking for the efficiency of the applied preparation process, ensuring access to materials relevant for submission to biodegradation tests.

**Figure 7:**
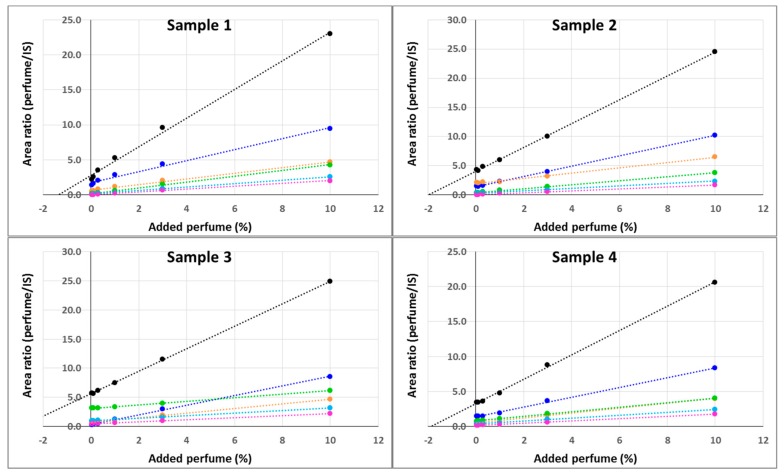
Standard addition quantification of Romascone^®^ (pink), Verdox^TM^ (green), Lorysia^®^ (light blue), Cyclosal (orange), Salicynile^®^ (blue) and the corresponding overall perfume (black) by Py-GC-MS for Sample 1 (upper left), Sample 2 (upper right), Sample 3 (lower left) and Sample 4 (lower right).

The results obtained using the external calibration curves were validated using the standard addition method. Solid samples were spiked with known amounts of perfume. It is important to point out that the quantity of internal standard present in each pyrolysis vial submitted to analysis was no longer constant, since the volume of calibration solution added depended on the amount of shells weighed (that cannot be a priori set in the required µg range). Therefore, for each measurement the area found was normalized based on the internal standard corrected by the ratio between the actual and theoretical volumes. The results obtained from the four different materials tested showed excellent linearity (Figure 7), with r^2^ ≥ 0.99 for both single ingredients and the whole perfume.

The values obtained using the standard addition and direct quantification methods are reported in Table 2. The two methods used gave results that are in good agreement. This is especially true considering that the typical biodegradation tests on polymers accept tolerances in values up to ±10%. Therefore, being able to determine a residual perfume quantity down to few percent is essential to be sure that the result of the test is not altered by this particular external factor.

The results in Table 2 are in contrast with what was obtained using the SPME-GC-MS method that systematically provided lower amounts of volatiles. This corroborates the hypothesis that perfumery molecules remained embedded in the polymeric shells even after extensive sample preparation. A destructuration method like Py-GC-MS has to be applied to access the total quantity of volatiles present in the polymeric material.

### 3.4. Application of Purification Procedure and Quantification Method to Biodegradation Test Samples

The application of the purification protocol and the validation of the samples using the Py-GC-MS method allowed a reduction of the variability on all samples tested for biodegradation with a global variability lower than 4%, on average. For replicates from the same test, the results were even better, with differences within 3% on average (OECD allows differences up to 20% between replicates). Indeed, the presence of residual perfume in the polymeric material erroneously resulted in higher biodegradation measurements (Table 3). The expected biodegradation of the shells in OECD screening tests was not higher than 5%, based on the biodegradation potential of the materials constituting it.

The importance of applying a reliable purification method including a control of the final purity of the polymeric material is of particular relevance to evaluate eco-friendly microcapsule candidates. This ensures access to reliable biodegradation results, which are critical to select prototypes with the potential to be optimized to reach satisfactory biodegradation levels.

It is clear that without a careful control of the material submitted to biodegradation tests, the results may be very different and misleading. False positive biodegradation results may lead to dramatic misinterpretations, risking bringing to the market microcapsules that are not at all eco-friendly.

## 4. Conclusions

A highly sensitive quantification method based on Py-GC-MS for residual volatiles in fragrance encapsulate polymeric materials was developed. This method allowed the development of a multistep purification protocol for polymers from complex samples like commercial slurries. The described method allows the quantification of residual volatiles that are suspected to be non-covalently embedded in the 3D polymeric network and therefore not quantifiable by other standard approaches (e.g., SPME-GC-MS or TGA). The methodology requires a very limited number of samples, it is applicable to virtually any volatiles and allows purity estimation above 99%. These characteristics make it ideally suited to control the purity of the polymeric material before further testing, in particular, biodegradation tests. Indeed, the impact of residual volatiles on the estimated biodegradability of fragrance encapsulates was demonstrated and should be taken into account to avoid misinterpretation of the potential biodegradability of encapsulating materials of the future.

## Figures and Tables

**Table 1:** Linear regression obtained by Py-GC-MS for each perfume ingredient and the corresponding total amount.

Raw Material	Equation	r^2^
Romascone^®^	y = 0.1621x + 0.0127	0.9996
Verdox^TM^ (main)	y = 0.2695x + 0.0296	0.9995
Verdox^TM^ (min)	y = 0.059x + 0.0008	0.9992
Lorysia^®^ (trans)	y = 0.1421x + 0.0169	0.9990
Lorysia^®^ (cis)	y = 0.0578x + 0.0015	0.9998
Cyclosal	y = 0.2832x + 0.0187	0.9996
Salicynile^®^	y = 0.5753x + 0.0167	0.9994
Overall perfume	y = 1.549x + 0.0953	0.9997

**Table 2:** Quantification of residual perfume in shells samples 1–4 using SPME-GC-MS and Py-GC-MS.

	Residual Perfume (%*_w_*_/*w*_)		
	SPME-GC-MS	Py-GC-MS	
		Direct Measure	Std Addition
Sample 1	1.09	1.45	1.36
Sample 2	0.38	1.07	2.00
Sample 3	0.18	2.16	2.92
Sample 4	N/A	1.23	1.88

**Table 3:** Comparison of biodegradation results of independent replicates for samples 1–3 with and without the application of the described purification protocol and validation of sample purity using Py-GC-MS quantification of residual volatiles.

	Biodegradation	
	TGA Guided Sample Preparation	Py-GC-MS Guided Sample Preparation
Sample 1		
Sample preparation 1	30%	3%
Sample preparation 2	26%	6%
Sample preparation 3	5%	7%
Sample preparation 4	11%	6%
Sample 2		
Sample preparation 1	22%	3%
Sample preparation 2	14%	4%
Sample preparation 3	18%	4%
Sample preparation 4	18%	5%
Sample 3		
Sample preparation 1	9%	1%
Sample preparation 2	14%	Not Available
Sample preparation 3	8%	Not Available
